# Respiratory Support in COVID-19 Patients, with a Focus on Resource-Limited Settings

**DOI:** 10.4269/ajtmh.20-0283

**Published:** 2020-04-21

**Authors:** Arjen M. Dondorp, Muhammad Hayat, Diptesh Aryal, Abi Beane, Marcus J. Schultz

**Affiliations:** 1 Mahidol Oxford Tropical Medicine Research Unit, Faculty of Tropical Medicine, Mahidol University, Bangkok, Thailand;; 2 Centre for Tropical Medicine and Global Health, Nuffield Department of Medicine, University of Oxford, Oxford, United Kingdom;; 3 Department of Intensive Care, Amsterdam University Medical Centers, Amsterdam, The Netherlands;; 4 Department of Anaesthesiology and Surgical Critical Care, Northwest General Hospital & Research Center, Hayatabad Peshawar, Pakistan;; 5 Department of Critical Care and Anesthesia, Nepal Mediciti Hospital, Lalitpur, Nepal

## Abstract

The ongoing novel coronavirus disease (COVID-19) pandemic is threatening the global human population, including in countries with resource-limited health facilities. Severe bilateral pneumonia is the main feature of severe COVID-19, and adequate ventilatory support is crucial for patient survival. Although our knowledge of the disease is still rapidly increasing, this review summarizes current guidance on the best provision of ventilatory support, with a focus on resource-limited settings. Key messages include that supplemental oxygen is a first essential step for the treatment of severe COVID-19 patients with hypoxemia and should be a primary focus in resource-limited settings where capacity for invasive ventilation is limited. Oxygen delivery can be increased by using a non-rebreathing mask and prone positioning. The presence of only hypoxemia should in general not trigger intubation because hypoxemia is often remarkably well tolerated. Patients with fatigue and at risk for exhaustion, because of respiratory distress, will require invasive ventilation. In these patients, lung protective ventilation is essential. Severe pneumonia in COVID-19 differs in some important aspects from other causes of severe pneumonia or acute respiratory distress syndrome, and limiting the positive end-expiratory pressure level on the ventilator may be important. This ventilation strategy might reduce the currently very high case fatality rate of more than 50% in invasively ventilated COVID-19 patients.

## INTRODUCTION

The novel coronavirus disease (COVID-19) pandemic, caused by the highly contagious severe acute respiratory syndrome-coronavirus-2 (SARS-CoV-2), is still at its height causing thousands of deaths each week. Since its start just a few months ago, our insights into the disease are rapidly increasing. Although several large randomized drug trials are underway, current survival from severe COVID-19 depends entirely on providing the best possible supportive care. Current recommendations for supportive care are mainly based on guidelines for the management of other viral pneumonias and sepsis. Yet COVID-19 seems to behave differently in some important aspects from other viral pneumonias and sepsis. In addition, recommendations tend to focus mainly on resource-rich settings,^1^ whereas recommendations for resource-poor settings in low- and middle-income countries, or for rich countries with health systems overwhelmed by the pandemic, are largely lacking. The management of COVID-19 has many aspects, and these have been summarized in some excellent preliminary and still evolving guidelines, in particular the WHO living document: https://www.who.int/docs/default-source/coronaviruse/clinical-management-of-novel-cov.pdf. We discuss here one of the most important components of the management of COVID-19, ventilatory support, with a special focus on resource-limited settings.

### Pathophysiology and histopathology of COVID-19.

Like the SARS coronavirus, SARS-CoV-2 enters the cell using the angiotensin converting enzyme-2 receptor, which is present on a wide range of human tissues, including the vascular endothelium (also in the lung), oral and nasopharyngeal mucosa, and type-II pneumocytes.^2^ A recent case series suggests two different pathophysiological patterns causing severe pulmonary injury.^3^ In patients with persistent high viral load in the lower and upper respiratory tract, pulmonary damage might be dominated by the direct cytopathic effect of the virus. An alternative pattern observed was a biphasic evolution with initial mild symptoms, followed by rapidly evolving late respiratory failure after 7–10 days of illness, despite a decreasing viral load, suggesting an immunopathological pathogenesis causing lung damage. This would agree with the observed high plasma concentration of pro-inflammatory cytokines later in the disease.^4,5^

Only a few histopathological studies on COVID-19 have been published to date, the majority in the Chinese language. These describe in early disease, obtained in patients dying from other causes, the presence of alveolar edema, proteinaceous exudates, and reactive pneumocyte hyperplasia, accompanied by mild inflammatory infiltration.^6^ In patients dying from COVID-19, the lungs show extensive alveolar proteinaceous and serous exudation, hyaline membrane formation, and inflammatory infiltration with multinucleated syncytial cells. In the alveolar space, the infiltrate contains monocytes and lymphocytes. Type-II alveolar epithelial cells show viral inclusion bodies, hyperplasia, as well as necrosis and desquamation. The lung microvasculature can show vascular edema and microthrombi. Parts of the lung can have alveolar exudate organization and pulmonary interstitial fibrosis.^7^ The presence of microthrombi fits with the observation that abnormal coagulation parameters, including increased plasma d-dimers, are associated with poor prognosis.^8^ Anecdotal evidence suggests that peripheral pulmonary thrombosis or embolism might be common in patients with severe COVID-19 (https://renal.org/wp-content/uploads/2020/04/COVID-19_synthesis-of-clinical-experience-in-UK-intensive-care_04.04.2020_FINAL.pdf). Of note, it is yet uncertain which of the histopathological pulmonary findings in fatal cases on ventilatory support are caused by the disease itself or are secondary to the injurious effects of invasive ventilation.

### Clinical and radiological features of COVID-19.

The clinical course of COVID-19 can range from asymptomatic to severe bilateral pneumonia eventually leading to death. Overall, an estimated 15% of patients with COVID-19 develop severe pulmonary involvement, but this proportion is highly dependent on the patient’s age and the presence of comorbidities such as obesity, diabetes mellitus, hypertension, and chronic pulmonary disease, and likely also renal disease.^5^ Estimated overall case fatality increases from an average 1.4% in patients younger than 60 years to 4.5% in those 60 years and older.^9^ After a mean incubation period of 5–6 (range 1–14) days, fever and a persistent severe dry cough are the most common initial symptoms. Fatigue, myalgia, arthralgia, sore throat, headache, nausea, and diarrhea are other frequent (from 5% to 40%) symptoms. Dyspnea and (severe) hypoxia are common reasons for admission to hospital, and these can gradually worsen during the course of the disease. An alternative pattern is that after a period of relatively mild symptoms, dyspnea and hypoxemia suddenly and quite rapidly worsen around day 10 of illness.^3,5,10^ In our personal experience, hypoxemia even less than an SaO_2_ of 90% is often remarkably well tolerated by COVID-19 patients, in particular in the age-group younger than 60 years, which is remarkably different from other causes of severe pneumonia and acute respiratory distress syndrome (ARDS).^11^ The extreme intrapulmonary shunt over the COVID-19 lesions can be responsible for the at times deep desaturations; patients experience relatively little dyspnea probably because of the persistence of spared, normal compliant lung tissue surrounding the affected areas. In principle, the respiratory system is the single organ failing in patients with severe COVID-19, which is different from patients with severe bacterial sepsis usually accompanied by multi-organ failure.

The chest X-ray in patients with moderate COVID-19 typically shows nonspecific multi-lobar infiltrates or pulmonary infiltration that can rapidly progress over 1–2 days. Chest computer tomography (CT) scan findings in COVID-19 patients are more specific, showing bilateral, multi-lobar, ground-glass opacification. With disease progression, these can become very extensive and complemented by increasing multifocal consolidative opacities surrounded by spared tissue. Another phenomenon observed in later stage disease is the so-called crazy paving, signifying thickened interlobular and intralobular lines in combination with a ground-glass pattern.^12,13^

### Management of COVID-19.

There is currently no proven antiviral treatment available for COVID-19. Based on in vitro activity against SARS-CoV-2, clinical trials evaluating hydroxychloroquine, chloroquine, remdesivir, lopinavir with ritonavir, and other compounds are planned or currently underway. The routine use of corticosteroids or other immunomodulatory therapies is not recommended at this moment. Availability and proper use of personal protective equipment (PPE) is essential to protect the frontline healthcare workers, as well as other patients without COVID-19. Recommendations on diagnostic strategies, treatment, and supportive care of patients with severe COVID-19 are covered in other documents^14^ (https://www.who.int/docs/default-source/coronaviruse/clinical-management-of-novel-cov.pdf). Worth highlighting is the routine use of low-molecular weight heparin, at prophylactic or higher doses, because of the hypercoagulative state in severe COVID-19, and a low threshold to start concomitant antibiotic treatment for secondary bacterial pneumonia. Restrictive fluid therapy may be important to avoid aggravation of pulmonary edema. Too stringent fluid restriction might contribute to renal dysfunction in patients with a compromised circulation caused by high-pressure invasive ventilation. It is currently unclear to what extend COVID-19 itself is an important cause of acute kidney injury.^15^

We here describe different modalities of respiratory support, with an emphasis on those feasible in resource-limited settings. Ventilatory support is essential for survival in patients with severe COVID-19, defined according to the WHO as the presence of oxygen saturation on air less than 93% by pulse oximetry, a respiratory rate above 30 per minute, or rapid progression of lung infiltrates on the chest X-ray, or critically ill COVID-19 patients with respiratory failure.

### Infrastructure for ventilatory support.

In 2015, the Lancet Commission on Global Surgery revealed that approximately one-quarter of hospitals surveyed in resource-limited countries lack sufficient oxygen supply.^16^ The most common modalities for oxygen supply include oxygen cylinders, oxygen concentrators, and centralized, piped oxygen systems, and the preferred modality will depend on local resources and supporting infrastructure.^17^ In the context of the high importance of oxygen therapy for patients with severe COVID-19, the WHO provides useful guidance on the different oxygen sources (https://www.who.int/publications-detail/oxygen-sources-and-distribution-for-covid-19-treatment-centres). Oxygen cylinders provide pressurized oxygen and require pressure regulators and flowmeters to deliver oxygen safely to the patient. Although easy to use, a common problem is oxygen leakage from ill-fitting adapters, which can cause up to 70% loss of the cylinder oxygen content.^18^ Oxygen cylinders will have to be replaced frequently, in particular when high oxygen flows are needed. This is not an issue using oxygen concentrators, which purify oxygen (> 90%) from ambient air through nitrogen-absorbing zeolite membranes. However, oxygen concentrators do need a continuous electrical power supply and require technical maintenance and regular filter changes. Not all models are suitable for use in tropical hot and humid environments, and poor maintenance can be an important issue.^19^ The WHO provides up-to-date information on oxygen concentrators and technical specifications to aid in the selection procurement and quality assurance (https://www.who.int/medical_devices/publications/tech_specs_oxygen-concentrators/en/). Most concentrators deliver oxygen flow rates of up to 6 L/min, which is sufficient to deliver oxygen noninvasively to moderately ill COVID-19 patients, but not to treat more severe hypoxia. Oxygen concentrators with a capacity up to 10 L/minute are increasingly available. Centralized, piped oxygen systems are used in better-resourced intensive care unit (ICU) settings. These deliver pressurized oxygen through wall outlets close to the ICU bed. The systems are usually fed by a large liquid oxygen tank or large oxygen cylinders. Engineering expertise and technical maintenance are essential for the proper functioning of centralized oxygen systems. Most modern mechanical ventilators use pressurized air and oxygen supply. Oxygen cylinders may be used for this purpose, but will require frequent exchange. Centralized, piped oxygen and air systems are thus the best option for running mechanical ventilators. Some mechanical ventilators do not require pressurized gas and generate their own driving pressure by internal air compressors. Oxygen concentrators can be used to enrich oxygen delivery by compressor-driven ventilators, although this will usually result in poorly defined inspiratory oxygen concentrations.

Blood gas analyzers assessing arterial blood oxygen and carbon dioxide (CO_2_) levels are used to titrate ventilatory support. However, these analyzers are expensive and require intensive maintenance. Availability of blood gas analyzers is severely limited in resource-limited settings^20^ and not needed for diagnosing ARDS.^21^ Pulse oximetry can be a cheap and reliable alternative, whereas in mechanically ventilated patients, end-tidal CO_2_ (etCO_2_) measurement might be a valid cheaper alternative for arterial CO_2_ measurements, although currently rarely available in resource-poor settings. Likewise, for lung imaging, lung ultrasound (LUS) could be an attractive and cheaper alternative to the chest X-ray or chest CT scan in COVID-19 (Dondorp and Schultz, in press). The Kigali modification of the Berlin definitions of ARDS uses pulse oximetry and LUS findings to define the severity of ARDS, and has proven to be an excellent alternative to the Berlin definition, which relies on blood gas analysis and chest X-ray or CT scan findings.^22,23^

### Supplemental oxygen.

In patients with moderate severe COVID-19, supplemental oxygen can be provided using simple nose prongs or face masks with an oxygen flow up to around 5–6 L O_2_/minute. Flow rates can be titrated using pulse oximetry monitoring, targeting an arterial oxygen content (SpO2) greater than 88%, which is a much more liberal target than in other causes of pneumonia. If the patient shows desaturation less than 88% for prolonged periods of time, oxygen delivery can be increased by using a non-rebreathing mask. These masks contain an additional reservoir bag where oxygen flows in, which is inhaled through a valve during inspiration. This can provide a fraction of inspired oxygen (FiO_2_) of 0.6–0.8 but will require an oxygen flow from the oxygen cylinder or piped oxygen of minimal 10 –15 L/minute. The use of non-rebreathing masks can be an important additional modality to increase oxygen delivery to patients with severe COVID-19, both in resource-rich and in resource-limited settings. In addition, patients can be nursed in the prone position or encouraged to lay on their front, which may give a remarkable improvement in oxygenation in COVID-19 patients (Schultz, personal communication). Sitting straight up can be an alternative, especially in patients for whom prone positioning is not feasible, for example, in severe obesity. Prone positioning facilitates ventilation of posterior lung field, improving the ventilation-perfusion mismatch, and thus oxygenation. High-flow nasal oxygen (HFNO) can also be used to importantly increase FiO_2_. Experience in the use of HFNO in coronavirus pneumonia is limited, and an important disadvantage for the resource-poor setting is the very high oxygen flow of up to 60 L/minute needed. Adult HFNO can either be delivered by the mechanical ventilator or by stand-alone systems, such as Optiflow^*R*^, which require a permanent power source, because they are often not battery-operated. This can be very dangerous in low-resourced settings when a permanent power supply cannot be guaranteed.

In patients treated with only supplemental oxygen, it is important to monitor fatigue or exhaustion because of increased work of breathing, in addition to monitoring oxygen saturation. A proportion of patients will not be sufficiently supported by just increasing FiO_2_. Using a non-rebreathing mask will not build up any positive end-expiratory pressure (PEEP), which is important to help preventing collapse of small airways and alveoli in the diseased lung at the end of expiration. As an intervention before invasive mechanical ventilation, PEEP can be generated by using continuous positive airway pressure (CPAP) or noninvasive ventilation (NIV) using biphasic positive airway pressure. Continuous positive airway pressure can be delivered with specific devices containing a PEEP valve providing resistance to exhalation, linked to a tight-fitting oral, nose, or full-face mask or a specific CPAP hood or helmet. Noninvasive ventilation requires a mechanical ventilator attached to a tight-fitting mask, and will in addition to PEEP also deliver additional inspiratory pressure to assist inspiration. Its use in patients with Middle East respiratory syndrome-related coronavirus showed a high failure rate of NIV, where it did not prevent intubation for ventilation.^24^ Currently, the recommendation is that HFNO, CPAP, or NIV in severe COVID-19 should only be used in selected patients with hypoxemic respiratory failure and that these patients are closely observed for early detection of further deterioration (https://www.who.int/docs/default-source/coronaviruse/clinical-management-of-novel-cov.pdf). In the current practice, these modalities are often used in patients where it is decided to forgo intubation for mechanical ventilation, for instance, because mechanical ventilation is not available, or in patients with a “do not resuscitate” directive. There is uncertainty around the potential for aerosolization when using HFNO, CPAP, or NIV, and these modalities should be used with airborne precautions until further evaluation of the safety is completed. An additional issue for resource-limited settings is that all these modalities require specific equipment, which can be expensive and difficult to procure in these times of a rapidly spreading pandemic. An exception could be CPAP. There are several initiatives for producing cheap CPAP helmets, which can be directly attached to an oxygen and compressed air cylinder.

### Invasive ventilation.

In many resource-limited settings, provision of quality mechanical ventilation is challenging. This is not only because of the low numbers of ICU beds equipped with mechanical ventilators but also because of issues related to infrastructure, equipment maintenance, human resources, and training. Concrete examples include the frequent need to reuse single-use components, poor access to consumables—including heat and moisture exchangers and suction catheters, poor access to spare ventilator parts like flow meters, unreliable oxygen supply, and inconsistent electricity.^25^ Novel coronavirus disease–specific infection prevention and control issues include the lack of close circuit endotracheal suction and absent or poor-quality heat moisture exchange filters with viral filtration capability. Particular in combination with poor availability of PPE, these shortcomings can pose an unacceptable risk to healthcare staff and should thus be addressed when countries aim for bolstering their mechanical ventilation capacity. Another important challenge, irrespective of the setting, is the rapid expansion of highly skilled capabilities needed for quality ICU care, for instance, to provide the complex ventilatory support needed in severe cases of COVID-19. This will require extended training to avoid that the intervention does more harm than good. Because of the shortage of ventilators, several centers, in particular in resource-limited setting, are contemplating the use of a single ventilator for multiple patients. Although an understandable emergency measure, this has unfortunately major disadvantages. For instance, this will result in unequal delivery of gas volumes and pressures to the individual patient, and will also compromise the individualized ventilator settings needed for optimal care. Given all these challenges and the high ICU mortality of COVID-19 in high-income countries, there is a strong argument for making effective oxygen provision the priority in low-resource settings.

The most appropriate timing for intubation of hypoxic patients with severe COVID-19 is not well known at the moment and will also depend on the local capacity for mechanical ventilation. Adoption of low-resource setting–specific triage algorithms might be appropriate.^26^ It seems that in a significant proportion of relatively younger patients, hypoxemia, even less than 88%, is reasonably well tolerated and not accompanied by severe respiratory distress or exhaustion. With the current experience, the trigger for intubation should, within certain limits, probably not be based on hypoxemia alone but more on respiratory distress and fatigue. Aerosols can be generated during intubation, and staff will need to wear N95, FFP2, or equivalent quality masks and take extra precautions to decrease the risks of infection. Intubation is preferably performed using a handheld video laryngoscope, as this allows for a larger distance between the mouth of the patient and the head of the doctor who intubates. However, video laryngoscopy will generally not be available in resource-limited settings.

Invasive ventilation can save lives in patients with severe respiratory distress. However, it can also aggravate or even cause damage,^27^ including barotrauma (air leaks, caused by high ventilation pressures), volutrauma (pulmonary edema, due to large tidal volumes), atelectrauma (repetitive opening and closing of vulnerable lung parts with atelectasis), biotrauma (local inflammation with spill to the systemic circulation of inflammatory mediators, bacteria, or bacterial products), and oxytrauma (by free oxygen radicals). In recent years, there is lesser emphasis on using higher PEEP to prevent atelectrauma.^28^ Mechanical ventilation in patients with critical COVID-19 differs in some important aspects from patients with ARDS from other causes. An important difference in COVID-19–affected lungs is the coexistence of severely affected lung areas adjacent to relatively unaffected areas. The affected areas with atelectasis are not, or very difficult, to open by recruitment procedures and higher PEEP. The unaffected areas remain remarkably compliant and are thus at risk of overdistension by higher PEEP levels. In these patients, strategies preventing atelectrauma by using higher PEEP could thus be harmful. This is analogous to the proposed tailored ventilation strategies according to ARDS phenotypes.^29^ Applying these phenotypes, COVID-19 seems to present primarily as “non-recruitable ARDS,” where high PEEP ventilation can cause ventilator-induced injury and increased mortality. Mechanical ventilation should aim to prevent damage caused by the ventilator by protecting undamaged and otherwise fragile lung tissue. This overrules the aims of achieving normoxaemia and normocapnia, allowing for permissive hypoxemia with PaO_2_ down to 8 kPa being acceptable and permissive hypercapnia with a pH down to 7.2 being acceptable.

### A strategy for invasive ventilation in COVID-19.

Following these principles, we suggest the following practical mechanical ventilation strategy. These suggestions might change when more evidence on the mechanical ventilation of COVID-19 patients will become available over time:

1. Use low tidal volumes. Limiting tidal volumes to 6 mL/kg ideal body weight (IBW) can reduce mortality by up to 25% in patients with ARDS.^30^ Guidelines strongly recommend to use such low tidal volumes without exception, in particular when the patient is receiving fully controlled ventilation (i.e., not only pressure support).^31,32^ The use of low tidal volumes of 6 mL/kg IBW, or even lower, can also be recommended strongly in patients with COVID-19.

When using low tidal volume ventilation, the following points are important to consider: 1) The IBW is not the same as the current, or actual body weight, and is calculated from the height of a patient; a simple calculation for a woman is “height (in cm) − 110” and for a man is “height (in cm) − 105.” 2) When using low tidal volumes, a higher respiratory rate, up to 35 per minute, should sometimes be accepted to achieve an adequate minute volume. This causes an increase in dead space ventilation, which can result in hypercapnia (“permissive hypercapnia”) or a slight deterioration in oxygenation. This can be permitted as long as the blood pH remains > 7.2 and SpO_2_ > 88%. 3) In case the ventilator is converted from fully controlled ventilation to pressure support ventilation, the tidal volumes can increase, sometimes greater than 6 mL/kg IBW. This can be accepted, provided the driving pressure remains low.

2. Use 10 cm H_2_O PEEP and be cautious using higher PEEP. Patients with ARDS need more PEEP than patients without ARDS, with a usual initial setting of 10 cm H_2_O. Positive end-expiratory pressure recruits collapsed lung areas and also keeps these partially open. A major drawback of PEEP is that it can also cause overdistension in more compliant parts of the lung. As stated earlier, this seems to be an important issue in COVID-19 patients. “Preventive high PEEP,” or “super PEEP,” with an intention to maximally recruit the lung parenchyma, is harmful and associated with excess mortality in patients with ARDS,^28,31^ and probably even more so in COVID-19 patients. This “open up the lung and keep the lung open” concept is often no longer used in patients with ARDS, and should probably not be used in COVID-19 patients. The concept is, however, still recommended in guidelines based on ventilation strategies for ARDS of other causes.

Regarding the use of PEEP, the following points are important to consider: 1) Applying higher PEEP may lead to better oxygenation, which can be falsely interpreted as a successful milestone of the intervention, because it signifies successful lung recruitment. However, it is important to remember that oxygenation is not the only goal of mechanical ventilation and that COVID-19 is characterized by areas of severely affected lung tissue that is not or hardly possible to open up. Recruitment through increasing PEEP should only be pursued if there is severe hypoxemia that does not respond to an increase in FiO_2_ greater than 0.6. 2) Too high PEEP (> 10 H_2_O) will cause overdistension of unaffected lung areas, which can result in an increase in driving pressure. 3) COVID-19 patients show next to the affected lung areas remarkably compliant unaffected lung areas, which can easily get overdistended. For this reason, the recommendation is to not apply more than 10 cm H_2_O PEEP and only use higher PEEP levels if this leads to a concomitant reduction in driving pressure. 4) Positive end-expiratory pressure levels greater than 10 cm H_2_O will compromise the circulation, especially if this causes overdistension of the lung; in case a patient with COVID-19 needs high doses of vasopressors to maintain an adequate blood pressure, the possibility of excessive PEEP should be considered. 5) Some patients on invasive ventilation may develop lung lesions over time, which are better recruitable. These patients might benefit from lung recruitment maneuvers and higher PEEP. A chest CT scan performed at two different PEEP levels (10 and 20 cm H_2_O) can be used to identify these lung areas, but this is impractical in patients with COVID-19 and often not available in resource-poor settings. Whether LUS can be used to distinguish recruitable lung lesions versus hyperinflation at higher PEEP levels is currently under investigation (Schultz, personal communication).

3. Monitor driving pressure. A high driving pressure is associated with poor patient outcome.^33^ The driving pressure is by approximation the difference between the maximum airway pressure (in pressure-controlled ventilation) or the plateau pressure (in volume-controlled ventilation) and the PEEP level. The driving pressure is not only an overall marker of lung damage but can also be iatrogenic resulting from too high tidal volumes or too much PEEP. There are, however, currently no randomized studies on interventions targeting the driving pressure.

The following points are important with regard to low driving pressures: 1) The easiest way to achieve a lower driving pressure is to limit the tidal volumes. 2) Adequate titration of PEEP may have a beneficial effect on the driving pressure; such titration requires experience, and can go either way: a decrease in PEEP can increase driving pressure (if resulting in increased lung atelectasis) or decrease it (if reducing lung overdistension), and the other way around. 3) Our current experience is that most COVID-19 patients can be ventilated with a low driving pressure less than 15 cm H_2_O, often as low as 5–7 cm H_2_O.

4. Use a low threshold for prone positioning. Prone positioning can improve oxygenation and improves patient outcome in ARDS.^34,35^ When applying prone positioning, the following practical points are important: 1) The sessions of prone positioning should be sufficiently long, that is, at least 16 hours or more per day.^36^ This means that the patient is kept only briefly—for a few hours—in the supine position. Within this regimen, the timing of turning the patient in the prone position and back can be flexible. 2) Studies in patients with ARDS not caused by COVID-19 show no difference in mortality between “responders” (patients who show an improvement in oxygenation in the prone position) and “nonresponders” (those who do not show this).^37,38^ Once prone position ventilation has been started, the decision on continuation of prone ventilation should not be based on the response observed in a single session. 3) Prone ventilation does not necessarily require additional sedation. Additional neuromuscular blockade in sedated patients is generally also not needed. However, some patients will start coughing when turned from a prone to supine position (or vice versa), and because of this, severe hypoxemia can develop. In these patients, a bolus of a neuromuscular blocking agent (after ensuring proper sedation) can be used to overcome this. 4) Related to the fact that in COVID-19 organ failure is usually limited to just the lung, sedative doses to achieve appropriate sedation are usually remarkably higher than in other intensive care patients. A combination of midazolam (or other short-acting intravenous benzodiazepines) and morphine can be used. If available, this can be supplemented with propofol if required to achieve sufficient sedation. However, because these patients require ventilation for a long time, it is wise to start weaning off sedatives early, especially in patients who develop multi-organ failure to prevent “rest-sedation” effects.

5. Weaning can be different. In COVID-19 patients, weaning from the ventilator and extubation might differ from patients with other forms of severe pneumonia or ARDS. Weaning and detubation can be facilitated by usually sustained muscle strength. However, COVID-19 patients may have continued thick and tenacious sputum production, which can result in a necessity for re-intubation. Whether these patients can benefit from a tracheostomy is yet unclear.

6. Good nursing care is essential. To cope with the high work load, many hospitals facing the pandemic organize support workers to assist the ICU nursing staff. Support workers can be trained and assist, for instance, in proning of patients. Some other important nursing aspects include regular and safe suctioning with clamping of the ventilator tubes to prevent circuit leak and unnecessary aerosol generation, bowel care to reduce constipation and bowel distension, and accurate management of the fluid balance.

Additional practical points include the following: 1) Maximize PEEP at 12 cm H_2_O, unless there is an important reason not to. 2) Set the targets for SpO2 at 88–92%, and set the etCO_2_ target allowing for hypercapnia accepting an arterial pH as low as 7.2. 3) COVID-19 patients show remarkably often a metabolic alkalosis with high plasma HCO_3_, for which the mechanism is not well understood. If indicated, this can be treated with oral acetazolamide. It should be realized that the metabolic alkalosis can be useful to compensate the respiratory acidosis in case of permissive hypercapnia as ventilation strategy.

## CONCLUSION

Oxygen therapy in patients with severe COVID-19 saves lives. Provisions for oxygen should be a global good. In resource-poor settings, assuring the availability of supplemental oxygen therapy should be a primary focus for the management of severe COVID-19. Oxygen delivery in COVID-19 patients with severe hypoxemia can be increased by using a non-rebreathing mask and prone positioning. The presence of only hypoxemia should within certain limits not trigger intubation because hypoxemia is often remarkably well tolerated. Patients with fatigue and at risk for exhaustion because of respiratory distress do require invasive ventilation. In these patients, lung protective ventilation is essential, for which limiting the PEEP level on the ventilator may be important. This might reduce the currently very high case fatality rate of more than 50% in invasively ventilated COVID-19 patients^39,40^ (https://www.icnarc.org/About/Latest-News/2020/04/04/Report-On-2249-Patients-Critically-Ill-With-Covid-19). Early experience from Amsterdam is that following this guidance case fatality in mechanically ventilated patients is less than 50% (Schultz, personal communication).

**Table:** 

Key messages on respiratory support for severe COVID-19: • Supplemental oxygen is essential for the treatment of severe COVID-19 with hypoxemia, also in resource-poor settings. • All hospitals coping with COVID-19 patients have to ensure the availability of supplementnal oxygen; oxygen sources include oxygen cylinders, oxygen concentrators, and centralized, piped oxygen systems. • Oxygen delivery can be increased by using a non-rebreathing mask and prone positioning. • Protection of healthcare workers with appropriate personal protective equipment is crucial, in particular during aerosol-generating procedures such as intubation, extubation, manual ventilation with a bag valve mask, or suctioning with opening of the ventilator circuit. • Continuous positive airway pressure (CPAP) or noninvasive ventilation with biphasic positive airway pressure (BiPAP) provides positive end-expiratory pressure (PEEP), but its position in the treatment of severe COVID-19 still needs to be defined better. • The presence of only hypoxemia should within certain limits not trigger intubation because hypoxemia is often remarkably well tolerated. • Patients with fatigue and at risk for exhaustion because of respiratory distress will require invasive ventilation. • In these patients, lung protective ventilation is essential, including a low tidal volume, permissive hypoxemia, and hypercapnia. • PEEP should be carefully titrated, and most patients can be managed with 10 cm H2O PEEP. • Driving pressures in COVID-19 patients are remarkably low – in case driving pressure rises, potential causes, like too high tidal volume, or too high or too low PEEP, should be addressed. • Invasive ventilation in the prone position should start early and last sufficiently long. • This ventilation strategy might reduce the currently very high case fatality rate of more than 50% in invasively ventilated COVID-19 patients.
